# Attitudes of Pediatric Nursing Staff Regarding Parental Presence During Cardiopulmonary Resuscitation

**DOI:** 10.7759/cureus.93037

**Published:** 2025-09-23

**Authors:** Dimitris Charalampopoulos, Theodoros Xanthos, Nicoletta Iacovidou, Dimitra Metallinou, Giannoula A Kyrkou

**Affiliations:** 1 Department of Infectious Diseases, Agia Sofia Children's Hospital, Athens, GRC; 2 Department of Midwifery, School of Health and Care Sciences, University of West Attica, Athens, GRC; 3 Department of Neonatology, National and Kapodistrian University of Athens, Aretaieion Hospital, Athens, GRC

**Keywords:** crna clinical training, family-centered care, healthcare ethics, nurses' attitudes, nursing care, parental presence, pediatric cpr, pediatric nursing, resuscitation team

## Abstract

This narrative review examines the attitudes of pediatric nursing staff toward parental presence during cardiopulmonary resuscitation (CPR), highlighting both international perspectives and findings from the Greek healthcare context. A thematic analysis of existing Greek and international literature was conducted using databases such as PubMed, Scopus, and Excerpta Medica Database (EMBASE), with studies selected based on predefined inclusion and exclusion criteria focusing on pediatric CPR, healthcare professionals’ attitudes, and the presence of parents during resuscitation. The studies reviewed support the inclusion of parents during pediatric resuscitation, reporting emotional, ethical, and psychological benefits for families. Pediatric nurses, in particular, tend to be more supportive of parental presence compared to their counterparts in adult care. This reflects a stronger alignment with family-centered care models. However, significant barriers remain, including institutional resistance, lack of formal guidelines, and insufficient staff training, especially in Greece. We also found that the majority of nursing staff reported having no personal experience with the presence of family during resuscitation and expressed disagreement with allowing parents to be present. Concerns about team performance and parental distress are also reported, though these are often mitigated by clear communication, staff preparation, and structured support roles. Overall, parental presence during pediatric CPR offers meaningful benefits when implemented within a supportive institutional framework. Targeted education, culturally sensitive communication, and the empowerment of pediatric nurses are essential to facilitate this practice. Adopting evidence-based policies and enhancing interdisciplinary collaboration can further promote ethically sound and family-centered resuscitation care.

## Introduction and background

Resuscitation refers to the set of urgent interventions applied to an individual when the heart and/or breathing have stopped, aiming to maintain blood circulation and oxygen delivery to the brain until the cardiac arrest victim achieves the return of spontaneous circulation (ROSC). For the safe and effective management of cardiac arrest victims, the presence of a resuscitation team is essential, and according to international guidelines, the role of healthcare professionals as members of this team is clearly defined.

Traditionally, the training of healthcare professionals in resuscitation has focused on technical skills and the knowledge required to provide optimal care. However, the effective functioning of a team also depends on non-technical skills such as communication, teamwork, leadership, task allocation, and situational awareness. Despite the critical and multifaceted role of nursing staff during resuscitation, their contribution is often overlooked, rendering them essentially “invisible” in the process. In recent years, resuscitation research has emerged as a dynamic and multifaceted field, attracting the interest of healthcare professionals and nurses worldwide. A large part of this research has focused on the knowledge and training of healthcare professionals in resuscitation, as well as on the retention and correct application of this knowledge for successful cardiopulmonary resuscitation (CPR) [1-36]. Most studies focusing on the training and knowledge retention of healthcare professionals concern adult resuscitation [1-26], while studies regarding pediatric resuscitation are more limited [28-35]. Even fewer studies have examined the effectiveness of pediatric and neonatal resuscitation training programs of the European Resuscitation Council (ERC) and the long-term retention of knowledge, with only two such studies identified [35,36].

Furthermore, while research has largely focused on technical aspects, the exploration of non-technical skills such as communication, collaboration, and the presence of family members during resuscitation remains limited. Factors such as workplace culture, assigned responsibilities, and education significantly influence these parameters. Most studies examining healthcare professionals’ attitudes focus on nursing staff involved in adult resuscitation [37].

Pediatric resuscitation poses additional challenges, and nurses’ attitudes toward family presence during resuscitation (FPDR) remain an underexplored area. Traditionally, the presence of parents was prohibited to prevent their emotional distress. However, recent evidence suggests that it can offer significant emotional, psychological, and ethical benefits to families. Allowing parents to be present during resuscitation can help them understand the events and accept the outcome, even in the case of death [38]. This practice aligns with the principles of family-centered care, which recognize the family as an integral part of the care process [39]. Nevertheless, its implementation largely depends on the attitudes and perceptions of the nursing staff. Pediatric nurses, in contrast to adult nurses, generally appear more supportive of parental presence [40]. However, concerns persist regarding the potential impact of parental presence on the resuscitation team's performance and the possible emotional burden on the parents themselves. These barriers are often associated with a lack of appropriate training and the absence of clear protocols [41,42].

Regarding the Greek context, a recent study revealed that healthcare professionals were unfamiliar with existing guidelines. Among other findings, 72.7% had not received relevant training, and 71.9% disagreed with allowing parental presence. These findings contrast with the international literature and indicate that the Greek healthcare system is at an early stage regarding the integration of the family in the resuscitation process [43].

Considering the above, it becomes evident that the attitudes of pediatric nurses toward parental presence during resuscitation constitute an issue of particular importance that remains largely unexplored, especially in the Greek context. The present study seeks to address this research gap by investigating the perceptions and attitudes of pediatric nurses regarding parental presence during resuscitation.

Aim

This literature review seeks to provide an overview and critical analysis of the attitudes of nursing staff, particularly pediatric nurses, toward parental presence during CPR, and to highlight the broader implications of this practice.

Objectives

This study seeks to investigate the specific attitudes of nurses, particularly pediatric nurses, toward parental presence during CPR. It also examines the reasons cited in the international literature both for and against this practice, evaluates the psychological and emotional impact of parental presence on both parents and nursing staff, and analyzes the implications of this presence for the effectiveness, communication, and dynamics of the resuscitation team.

## Review

Methods

Study Strategy

The present literature review was conducted through a comprehensive search of both international and Greek scientific literature. The search was performed in May 2025 across the PubMed, Scopus, and Excerpta Medica Database (EMBASE) databases. The following keywords and their combinations were used: “pediatric CPR,” “parental presence,” “nurses’ attitudes,” “family-centered care,” “pediatric nursing,” “resuscitation team,” “training and education,” “nursing care,” and “healthcare ethics.” Given the limited number of available studies on this topic, no publication date restrictions or geographical filters were applied to the search strategy.

Inclusion Criteria

The review will include original research articles, systematic reviews, and qualitative studies that provide insights into attitudes, experiences, and perceptions related to parental presence during CPR. Eligible studies must be published in English or Greek and available in full text through academic databases. The focus of the research should be on pediatric CPR, particularly addressing the attitudes, perceptions, experiences, or impacts associated with the presence of parents during resuscitation. Studies conducted in adult CPR settings will also be considered if they offer a comparative perspective relevant to pediatric contexts. Finally, the review will include studies involving healthcare professionals, with a primary emphasis on pediatric nurses, as well as parents.

Exclusion Criteria

Studies will be excluded if they do not directly address parental presence during CPR or the attitudes of healthcare professionals toward this practice. In addition, research focusing exclusively on populations other than healthcare professionals, nurses, or parents, or on groups not associated with pediatric care settings, will be excluded unless such studies provide comparative insights that are particularly relevant to the pediatric context.

Results

Given the aim of the present study to explore existing literature on the subject, a comprehensive literature review was conducted. The articles included in the review presented considerable heterogeneity, both in terms of study samples (as different population groups were examined) and in the methodologies employed. Due to these variations, it was not feasible to conduct a systematic review or meta-analysis, consistent with the 2023 International Liaison Committee on Resuscitation (ILCOR)/International Consensus, which notes that the heterogeneity of studies on FPDR precludes meta-analysis (ILCOR, 2023) [44]. Instead, a qualitative synthesis of the data was performed using thematic analysis. The selected articles were grouped into thematic categories, dominant patterns were identified and coded, and concise conclusions were drawn for each thematic unit. This approach allowed for a structured understanding of the key findings and trends emerging from the literature.

Child's Needs

Pediatric nursing constitutes a specialized branch of nursing science, focused on providing holistic care to infants, children, and adolescents, taking into account their developmental, psychological, and social specificities. A child needs to be able to communicate with those around them, to be understood, to feel safe, and for their physical needs to be met. Pediatric nurses must recognize these needs and respond promptly to meet them.

In a 2016 study, it is stated: "Children recognize the role of nurses in the care they receive during hospitalization and the importance of this for their recovery. For children, nursing care is directly related to discharge from the hospital. Therefore, they request that this care be carried out correctly and promptly to improve their health or recover and return home. Children also emphasize the importance of providing this care with tenderness, affection, and respect. Additionally, they need to have each step of the procedures explained so that they understand their purpose and feel safer. They said that sometimes simple procedures end up causing pain, which requires greater attention and sensitivity from the nurse. Play was recognized as a way to minimize stress and fear during these times. This study showed the importance of a child’s perspective on the care they receive" [45].

In another study, it was found that "Children showed a high level of trust in the nursing staff. The trust of children and parents in the staff was found to be related, while the gender of the nursing staff did not affect their trust. Younger children had higher levels of trust than older children, particularly in the honesty of the nurse. This trust was diminished if the child had previous hospitalizations. One-fifth of children expressed fear toward the nursing staff. A child who trusts is likely to be less fearful. Pediatric nurses should recognize that trust can change with age and with multiple hospitalizations" (Table 1) [46].

**Table 1:** Visualization of the child’s need and the pediatric nurse’s role This table presents key child needs during care and the corresponding pediatric nurse’s role in meeting them through communication, safety, comfort, and empathy [45,46].

Child's Need	Pediatric Nurse's Role
To communicate	Actively listen, use age-appropriate language, and ensure understanding.
To be understood	Pay attention to verbal and non-verbal cues, and seek clarification when needed.
To feel safe	Provide reassurance, explain procedures, and create a calm and supportive environment.
Physical needs to be met	Provide necessary medical care, ensure comfort, and manage pain effectively.
To understand procedures	Explain each step clearly and simply, and answer questions honestly.
To receive care with tenderness, affection, and respect	Demonstrate empathy, provide gentle care, and acknowledge their feelings.

Ethical Values of the Nurse

The ethical values held by healthcare professionals involved in pediatric care significantly influence their attitudes and practices. These values are shaped by religious and social beliefs, personal experiences, and empathy. Fundamental ethical principles in pediatric care encompass justice, non-maleficence, autonomy, beneficence, dignity, and integrity [47]. The unique nature of the parent-child relationship, alongside cultural differences, profoundly impacts the provision of care, necessitating that the family be regarded as a cohesive unit [40]. Institutional policies and nurses’ convictions regarding family involvement play a critical role in determining the extent to which families are integrated into daily patient care [48]. Pediatric palliative nursing care, while emotionally demanding and challenging, is also highly rewarding, underscoring the imperative for further research to better understand and address the needs of terminally ill pediatric patients, their families, and the nurses who care for them [49].

Parental Presence During Resuscitation

Is the presence of parents during resuscitation beneficial? Does it contribute to the successful outcome of CPR? Does it help parents better accept the loss of their child? How do nurses react to this presence? How do they feel? Does it influence their attitudes?

The primary goal of intervention during resuscitation is to achieve a successful outcome and to mitigate the negative consequences of loss. The death of a child is a deeply unnatural event, disrupting the expected flow of life. The grieving period in such cases is often prolonged and has been associated with increased parental mortality, as highlighted by a large cohort study conducted in Denmark (Li et al., 2003) [50]. This study analyzed data from national registries and demonstrated a link between child loss and increased mortality among parents. However, it did not identify any direct association between parental presence during resuscitation and increased mortality. It is likely that other factors, particularly psychosocial, contribute significantly to these outcomes.

A qualitative study by Stewart (2019) [51], based primarily on semi-structured interviews with parents who had experienced their child’s resuscitation, revealed that parents often perceive a sense of overwhelming chaos. Nevertheless, they simultaneously express an innate need to be present and to know what is happening. While emotional support is appreciated, physical presence and the ability to receive real-time updates from the healthcare team are considered even more important. Parents also value the sense that the resuscitation team is personally invested in their child's care. This study underscores the significance of emotional connection and continuous communication with healthcare staff, elements often absent from quantitative research.

A systematic review by Shaw et al. (2011) [52], incorporating both qualitative and quantitative studies, concluded that parental presence is a controversial issue. However, most parents believe they should have the right to choose whether to be present during resuscitation. The review also supports the view that while it is acceptable to ask parents to leave if their presence is detrimental to the child’s care, the overall benefits of parental presence tend to outweigh potential harms. It may help parents come to terms with the death of their child. Nevertheless, the heterogeneity of methods and sample sizes in the primary studies limits the reliability and generalizability of conclusions. Similar findings are reported by Tinsley et al. (2008) [38].

In a review, McAlvin (2014) [53] argues that family presence should be encouraged. Trained healthcare professionals should act as liaisons between the family and the resuscitation team. Importantly, these professionals must not be directly involved in the resuscitation efforts. Their role must be predetermined and based on specific protocols. Parents who were present during the procedure reportedly coped better with their child’s death. The recommendations in this review are based on a systematic analysis of the international literature.

A 2023 study by Brunt & Alnababtah [54] reinforces these conclusions, showing that parents report more positive experiences when they are allowed to choose their level of presence and involvement during resuscitation.

In 2022, a qualitative study by Ghavi et al. [55] provided further insights. The authors stress that parents must be adequately prepared before witnessing resuscitation. They should receive frequent and confidential updates and must be supported empathetically by a healthcare professional dedicated to their needs. Most importantly, cultural context, psychological state, and spiritual beliefs must be considered. The study recommends that future research explore parental readiness across different cultures, as needs and expectations may vary. The authors emphasize the importance of delivering information with both empathy and a balance between hope and despair.

However, to implement such approaches effectively, healthcare professionals must receive appropriate training. As Ferreira et al. (2014) [41] note, introducing values, behaviors, and attitudes that promote FPDR requires the development of institutional policies and awareness programs. These programs must involve the family and aim to meet their emotional and informational needs.

The necessity of educational programs is also emphasized in a study by Gutysz-Wojnicka et al. (2018) [42], who state: “A positive experience supports a nurse’s beliefs and attitudes. The process of changing attitudes towards family presence requires positive work environments where healthcare professionals can gain supportive experiences.” Key factors influencing attitudes toward family presence include the introduction of strategic training programs and confidence-building measures.

When examining the attitudes of nurses regarding FPDR, Fulbrook et al. (2007) [39] concluded: “The majority of pediatric intensive care nurses support parental presence during CPR. While concerns exist about potential negative effects on both parents and the resuscitation team, these are largely outweighed by the commitment to doing what is best for the child and family. Pediatric nurses appear to be more supportive than adult-care nurses, reflecting the broader adoption of family-centered care models in pediatric hospitals.”

A Greek study by Vavarouta et al. (2011) [43] examined the attitudes of medical and nursing personnel regarding parental presence during resuscitation and other invasive procedures. The findings revealed that 73.6% of participants were not familiar with current guidelines and recommendations, while 72.7% had not received relevant training. In addition, 76.9% reported having no personal experience with FPDR, and 71.9% expressed disagreement with allowing parental presence. Differences were also observed between professional groups: 43.2% of physicians and 14.3% of nurses believed that the decision should rest solely with doctors, whereas 40.3% of nurses and 20.5% of physicians favored a shared decision-making approach.

The healthcare personnel in Greece appear largely unfamiliar with the practice of family presence, leading the authors to recommend the implementation of educational programs, along with institutional guidelines and policies.

Discussion

Child's Needs

Nursing is the science of care, but pediatric nurses are not simply care providers. Findings show that pediatric nursing is multidimensional and requires understanding the unique developmental, emotional, and psychological needs of children. Nurses act as communication bridges between the child and their environment, provide emotional support, and play a key role throughout hospitalization and treatment. Children recognize care and associate it with recovery, so care must be delivered with sensitivity and respect.

Communication should be age-appropriate, explaining procedures clearly and honestly to help children feel safe. Play and distraction techniques, suggested by the children themselves, help reduce anxiety. Trust is fundamental in the nurse-child relationship, influenced by age and experience, requiring nurses to adjust their behavior accordingly. The nurse’s gender does not affect trust; communication quality and transparency are what matter most.

Ultimately, the child’s voice must be heard, and they should participate in planning their care. Empathy, trust, and effective communication form the foundation of modern pediatric nursing [45,46].

Ethical Values of the Nurse

The attitudes and behaviors of those involved in pediatric care are shaped by ethical values influenced by religious beliefs, personal experiences, social norms, and empathy within a sociocultural context. Healthcare professionals’ ethics are guided by both personal morals and external cultural factors.

Beauchamp et al. outline core ethical principles: justice, non-maleficence, autonomy, beneficence, dignity, and integrity, all crucial in pediatric care [47]. Justice ensures equal yet individualized care; non-maleficence requires avoiding harm, especially in high-risk interventions like resuscitation. Autonomy respects the child’s growing decision-making ability, beneficence promotes the child’s well-being, and dignity and integrity acknowledge each child’s inherent worth.

A 2004 comparative study across Australia, Indonesia, the UK, and Thailand emphasized recognizing the parent-child bond during hospitalization and treating the family as a unit in care [40]. This approach respects cultural backgrounds and supports parental involvement, which improves outcomes. Therefore, nursing education and healthcare systems must integrate transcultural knowledge while upholding core nursing ethics.

Pediatric care must adapt ethical values to both universal principles and cultural norms to provide individualized care. Healthcare professionals need training to apply ethics in diverse settings, protecting the child’s best interests and respecting family values.

Parental Presence During Resuscitation

Parental presence during pediatric resuscitation remains a complex and emotionally charged issue, interwoven with cultural, ethical, and psychological dimensions. The literature reveals a wide spectrum of opinions, ranging from strong support to marked reservations. These variations are influenced by cultural and societal norms, the level of healthcare professionals' training, and the emotional readiness of both parents and healthcare staff.

The primary goal of resuscitation is the child’s survival and the minimization of trauma for both the family and the medical team. However, the impact of parental presence on the outcomes of resuscitation is not clearly established. Nevertheless, an increasing body of evidence suggests that parental presence may facilitate coping, regardless of the clinical outcome. Studies by Stewart (2019) [51] and Tinsley et al. (2008) [38] indicate that witnessing the resuscitation process helps parents understand what transpired and may support the grieving process in the event of loss.

It appears, however, that emotional support alone is insufficient if not accompanied by simultaneous, clear communication regarding the resuscitation process. This is consistent with principles of family-centered care, which prioritize communication, respect, and the recognition of the family’s needs and choices. Within this framework, the findings of Shaw et al. (2011) [52] and Brunt & Alnababtah (2023) [54] are particularly significant. Parents who are present and continuously informed during resuscitation tend to report more positive experiences and a greater sense of closure.

Healthcare professionals, however, are not in full agreement regarding parental presence during resuscitation. McAlvin (2014) [53] and Ferreira et al. (2014) [41] advocate for the implementation of structured protocols and the involvement of specially trained support personnel. In practice, however, such systems, which require institutional commitment and alignment with healthcare policies, as well as staff training, are rarely implemented. Gutysz-Wojnicka et al. (2018) [42] emphasize the need for systemic and institutional changes to shift healthcare providers' attitudes. Positive personal experiences by staff members also play an important role in this transformation.

Pediatric nurses, in particular, appear to be more sensitized and generally exhibit a more favorable stance toward parental presence, as shown in Fulbrook et al. (2007) [39]. This may be attributed to the prevailing culture in pediatric care settings, where family-centered models of care are more commonly adopted and valued.

Equally important is the consideration of the cultural, spiritual, and emotional dimensions of parental presence. As noted by Ghavi et al. (2022) [55], preparation, empathy, and culturally sensitive communication are critical. Personalized approaches that recognize the diversity in expectations and readiness among parents are essential for their safe and supportive integration during resuscitation events.

The study by Vavarouta et al. (2011) [43] significantly differs from others, both methodologically and contextually. It utilizes a structured questionnaire with closed-ended questions, in contrast to semi-structured interviews [50,55], open-ended surveys (Brunt), and literature reviews [52,53]. It also focuses on the perspectives of healthcare professionals in Greece, whereas other studies concentrate on parental experiences [38,51,55], healthcare providers' attitudes [39,42], or implementation strategies [41,53]. Notably, the sample size in Vavarouta’s study [43] is relatively large (216 healthcare professionals, physicians, and nurses from Greek hospitals) compared to other studies with smaller, qualitative samples (e.g., ≤30 parents in Stewart & Ghavi) [51,55] larger international cohorts (e.g., >500 in Fulbrook) [39], or studies based on systematic review methodology. Importantly, it is one of the few studies in the Greek context focusing exclusively on healthcare professionals’ attitudes without examining parental experiences. In contrast, most recent international studies adopt qualitative designs focusing on parents’ lived experiences and actively advocate for family inclusion. While international research explores the emotional, ethical, and cultural complexities of parental presence during resuscitation, the Vavarouta study [43] is limited to recording professionals' knowledge, attitudes, and experiences, highlighting a clear gap in training and institutional guidance.

Unlike international data, Greek studies report significantly lower levels of support for parental presence during CPR, primarily due to the lack of awareness and training among nurses in Greece. Therefore, the Greek reality appears to be at an early stage in integrating family involvement in the resuscitation process. This underscores the need for institutional reforms, professional training, and stronger support structures for families during CPR.

Parental presence during CPR undoubtedly influences the dynamics and operation of the resuscitation team. While some professionals express concerns that it may distract team members or increase their stress levels, evidence shows that when resuscitation protocols are followed and staff are adequately trained, parental presence can be successfully integrated without disrupting team performance.

McAlvin (2014) [53] and Ferreira et al. (2014) [41] highlight the importance of assigning healthcare professionals specifically to support the family, separate from the resuscitation effort. This approach avoids interference with clinical procedures while maintaining communication with the family.

In summary, parental presence does not appear to disrupt team dynamics when appropriate structures, guidelines, and staff training are in place. We should take into account that the parental presence during resuscitation may not directly influence clinical outcomes, but it has considerable emotional and psychological benefits for families. At the same time, it presents challenges for clinical teams, particularly in environments lacking formal training and established protocols. Future efforts should prioritize the development of evidence-based guidelines, comprehensive staff training, and the promotion of organizational cultures that value and support family-centered care. Continued research, particularly in non-Western settings, is essential to better understand the complex needs and preferences of families across diverse sociocultural contexts.

This synthesis of studies with diverse methodological approaches, including qualitative interviews and cohort studies to systematic reviews, provides a holistic perspective on the issue of parental presence during pediatric resuscitation. Despite the heterogeneity of evidence, the majority of findings support the emotional benefits for parents and the ethical imperative of offering them the right to choose. Successful implementation of family presence depends on staff training, institutional support, and culturally sensitive communication strategies. Future research should address cultural variation and aim to establish evidence-based, standardized protocols.

Parental presence during pediatric CPR is not merely an act of compassion; it is a component of high-quality, family-centered, and safe healthcare (Topjian et al., 2020; Van de Voorde et al., 2021). Institutional policies, the empowerment of the nursing role, and ongoing education are essential for ensuring the effective implementation of this practice across diverse cultural and healthcare contexts. International cooperation and the exchange of best practices can serve as a catalyst for universal adoption and cultural integration of parental presence in resuscitation protocols (Table 2) [56,57].

**Table 2:** Policy and educational strategies for supporting parental presence during pediatric cardiopulmonary resuscitation (CPR) This table summarizes key intervention areas, specific proposals, and objectives aimed at promoting parental presence during pediatric CPR through policy, education, collaboration, and public engagement [38-55]. AHA: American Heart Association; ERC: European Resuscitation Council

Intervention Area	Proposal	Objective
Regulatory framework & guidelines	Development of national or institutional guidelines aligned with international recommendations (AHA, 2020; ERC, 2021), explicitly supporting parental presence [56,57]	Safe, evidence-based, and consistent implementation
Professional education of nurses	Design of accredited educational programs that include communication skills, emotional regulation, and cultural competence training	Strengthening nurses’ self-efficacy and preparedness
Continuing professional development	Ongoing education on updated CPR guidelines and family-centered care through interactive workshops and simulations	Keeping professionals informed and skilled
Interdisciplinary collaboration	Fostering a culture of collaboration between doctors and nurses through joint training and shared clinical protocols	Enhancing team communication and shared decision-making
Nursing role empowerment	Recognizing nurses as key decision-makers and family advocates during resuscitation events	Promoting professional autonomy and responsibility
Documentation & research	Supporting international and local research on professionals’ and families’ perceptions and experiences with parental presence	Informing evidence-based policy and clinical practice
Public awareness and engagement	Creating educational campaigns and resources for the general public and caregivers about the benefits of family presence during CPR	Reducing resistance and increasing societal acceptance

Limitations

Due to the significant heterogeneity in study samples and methodologies in the included articles, it was not possible to perform a systematic review or meta-analysis. This approach means that the study may be vulnerable to selection bias and lack the rigor for quantitative synthesis, potentially limiting the generalizability and definitiveness of its conclusions.

Furthermore, the only Greek study included highlights that Greek healthcare professionals show limited awareness and education regarding parental presence, suggesting that findings relevant to the Greek context may reflect an early stage of mainstreaming of family involvement compared to international practice.

## Conclusions

Parental presence during pediatric CPR is a complex yet beneficial practice. It supports families in managing loss and enhances their sense of control, while also having a positive influence on the dynamics of the healthcare team. Nevertheless, decision-making often remains primarily a medical privilege, with nurses mainly confined to a mediating role rather than active participation.

Pediatric nurses generally demonstrate stronger support for parental presence, in line with family-centered care approaches. However, limited training and a lack of institutional support create barriers and uncertainty in implementing this practice. Strengthening education, establishing clear guidelines, and fostering interdisciplinary collaboration are essential for the development of ethically sound and genuinely family-centered resuscitation care.
